# Anti-inflammatory properties of ursodeoxycholyl lysophosphatidylethanolamide in endotoxin-mediated inflammatory liver injury

**DOI:** 10.1371/journal.pone.0197836

**Published:** 2018-05-24

**Authors:** Johannes Maximilian Ludwig, Yuling Zhang, Walee Chamulitrat, Wolfgang Stremmel, Anita Pathil

**Affiliations:** 1 Department of Internal Medicine IV, Gastroenterology and Hepatology, University of Heidelberg, Heidelberg, Germany; 2 Department of Diagnostic and Interventional Radiology and Neuroradiology, University Hospital Essen, Essen, Germany; University of Navarra School of Medicine and Center for Applied Medical Research (CIMA), SPAIN

## Abstract

**Aim:**

Endotoxin-mediated liver inflammation is a key component of many acute and chronic liver diseases contributing to liver damage, fibrosis and eventually organ failure. Here, we investigated ursodeoxycholyl lysophosphatidylethanolamide (UDCA-LPE), a synthetic bile acid-phospholipid conjugate regarding its anti-inflammatory and anti-fibrogenic properties.

**Methods:**

Anti-inflammatory properties of UDCA-LPE were evaluated in a mouse model of D-galactosamine/lipopolysaccharide (GalN/LPS)-induced acute liver injury, LPS treated RAW264.7 macrophages and murine primary Kupffer cells. Furthermore, anti-inflammatory and anti-fibrotic effects of UDCA-LPE were studied on primary hepatic stellate cells (HSC) incubated with supernatant from LPS±UDCA-LPE treated RAW264.7 cells.

**Results:**

UDCA-LPE ameliorated LPS-induced increase of IL-6, TNF-α, TGF-β, NOX-2 in the GalN/LPS model by up to 80.2% for IL-6. Similarly, UDCA-LPE markedly decreased the expression of inflammatory cytokines IL-6, TNF-α and TGF-β as well as the chemokines MCP1 and RANTES in LPS-stimulated RAW 264.7 cells. Anti-inflammatory effects were also observed in primary murine Kupffer cells. Mechanistic evaluation revealed a reversion of LPS-activated pro-inflammatory TLR4 pathway by UDCA-LPE. Moreover, UDCA-LPE inhibited iNOS and NOX-2 expression while activating eNOS via phosphorylation of AKT and pERK1/2 in RAW264.7 cells. HSC treated with conditioned medium from LPS±UDCA-LPE RAW264.7 cells showed lower fibrogenic activation due to less SMAD2/3 phosphorylation, reduced expression of profibrogenic CTGF and reduced pro-inflammatory chemokine expression.

**Conclusion:**

In the setting of endotoxin-mediated liver inflammation, UDCA-LPE exerts profound anti-inflammatory and anti-fibrotic effect implying a promising potential for the drug candidate as an experimental approach for the treatment of acute and chronic liver diseases.

## Materials & methods

UDCA-LPE synthesis was performed by ChemCon (Freiburg, Germany). All other chemicals were obtained from Sigma (Munich, Germany) unless stated otherwise.

### Cell cultures

Mouse macrophage cell line RAW 264.7 (a gift from Prof. A. Dalpe) was cultured in DMEM Medium (PAA Laboratories GmbH, Germany) supplemented with 4,5g/l Glucose, 2 mM L-Glutamine and 10% FCS (Gibco^®^, USA). Primary human hepatic stellate cells (Innoprot, Spain; Cat.-Ref.: P10653;) were cultured with a stellate cell medium kit (Ref.: P60126) as instructed by the manufacturer. Cell culture flasks for HSC were pretreated with Poly-L-Lysin (Innoprot, Spain; Cat.-Ref.: PLL). For treatment, UDCA-LPE was solubilized in ethanol (20 mM), LPS (Cat.-Ref.: L2880-10MG) was reconstituted in PBS (1 mg/ml), Interleukin-6 (R&D Systems, Inc., USA Cat.Ref.: 206-IL-010) was solubilized in 0.7% BSA supplemented PBS (20 μg/ml), and TGF-β was prepared in a 4 mM HCL + 1 mg/ml BSA solution (1 μg/ml). Control cells were incubated with the same solvent of each agent correspondingly. Treatment with UDCA-LPE (50/90 μM) was started 30 min. prior to treatment with LPS (500 ng/ml), IL-6 (10 ng/ml) or TGF-β (4 ng/ml). For generation of conditioned RAW264.7 Medium, cells were treated for 24h prior to transferring the conditioned supernatant onto HSC cells after removing cells and cell fragments by centrifugation.

### Mouse model

Liver failure induction and treatment was performed as previously described [1]. Briefly, male C57BL/6 mice (Charles River Laboratories, Sulzfeld, Germany) at the age of 8 weeks received i.p. injections of Galactosamine (GalN; 700 mg/kg) and LPS (10 μg/kg) dissolved in PBS to induce an acute liver failure. UDCA-LPE was dissolved in 0.5% carboxy-methylcellulose in PBS and mice were treated with 30 mg/kg i.p. 1 h prior to GalN/LPS challenge. Control animals received injections of drug solvents only. Euthanasia was performed 5 h after treatment start with GalN/LPS. During this time no mortality was observed. Liver tissue was harvested and snap frozen in liquid nitrogen and stored at -80 °C until analysis. All performed experiments were approved by the Animal Care and Use Committee of the University of Heidelberg.

### Immunoblotting

Western blotting was performed as previously described [2]. Briefly, cell lysates (15–50 μg protein) were separated by gel electrophoresis and blotted onto a PVDF membrane. Incubation with primary antibodies was performed at 4 °C overnight while staining with secondary antibodies was performed for 1h at room temperature. A list of the used antibodies and dilutions is provided in the S1 and S2 Tables. Protein bands were visualized via Luminata Forte ECL system (Merck Millipore, Germany) and Amersham Hyperfilm^™^ (GE Healthcare limited, USA) X-ray film. Protein band density was quantified utilizing the Image J software (NIH; https://imagej.nih.gov/ij/)). Results from statistical evaluation can be found in the supporting information document.

### Gene expression analysis by quantitative real-time PCR (qRT-PCR)

TaqMan Gene Expression Assays (Applied Biosystems, Germany) were used as recommended by the manufacturer. Specific details on RNA-isolation from liver tissue and cell culture cells, DNA Synthesis and performing the qRT-PCR are described in the supporting document.

#### Nitric oxide quantification

Nitric oxide in treatment medium supernatant was quantified after 6h of treatment via the Griess reaction using the Griess Reagent Kit for Nitrite Determination (Invitrogen™, USA) according to the manufacturer´s protocol. The selective inducible nitric oxide synthase (iNOS) inhibitor W1400 (Biomol, USA) was reconstituted in methanol (40 mM). Cells were pre-incubated with 25 μM W1400 for 5 min. prior to treatment with LPS ± UDCA-LPE.

### Enzyme linked immunosorbent assay (ELISA)

Interleukin-6 (IL-6) in treatment medium supernatant was measured using the Duo-Set^®^ Mouse IL-6 (DY406) (R&D-Systems, USA) according to the manufacturers protocol. 10% BSA solution, developer solution and stopping solution were purchased from SABiosciences Corp., USA.

### Statistical data analysis

Groups were compared using the unpaired two-tailed student´s t-test for comparing two groups. One-way ANOVA including Bonferoni post-hoc test was used for comparing three or more groups using GraphPad Prism 5.0 for Mac (GraphPad Software, Inc, USA). All error bars represent the standard deviation of the mean (SD) unless stated otherwise. A p-value <0.05 was considered statistically significant.

Note: Additional methods are described in the supporting information document.

## Introduction

Liver inflammation is a key component in many acute and chronic liver diseases leading to parenchymal damage and progressing to fibrosis, liver cancer and eventually to liver failure [3, 4]. Regardless of the etiology, the activation of the innate immune system via Toll-Like receptor-4 (TLR4) signaling pathway through exogenous ligands such as e.g. bacterial lipopolysaccharides (LPS) and endogenous damage associated ligands stays at the center of many liver inflammatory diseases such as e.g. nonalcoholic (NASH) and alcoholic (ASH) steatohepatitis, oxidative drug toxicity, hepatic immune disorders, and liver allograft rejection [4].

Within the liver, TLR4 is expressed on all cells but shows the highest expression on resident liver macrophages, the Kupffer cells (KC), which after TLR4 pathway activation primarily drive the inflammatory cascade by secretion of pro-inflammatory cytokines and chemokines, production of reactive oxygen species (ROS), and synthesis of nitric oxide (NO) leading to dysregulation of liver homeostasis [5–7]. Moreover, within this inflammatory environment, quiescent hepatic stellate cells (HSC) undergo activation, facilitate the inflammatory reaction and ultimately cause liver fibrosis by secretion pro-fibrogenic growth factors such as connective tissue growth factor (CTGF) and extracellular matrix. The key roles of KC and HSC in inflammatory liver disease and the fact that depletion of these cells greatly limits liver inflammation and fibrosis in experimental models makes them an ideal pharmacological target for treatment of inflammatory liver diseases [8, 9].

Phospholipids such as phosphatidylcholines (PC) are present in all mammal cells and are crucial for the maintenance of membrane integrity and protection against apoptosis and inflammation [10, 11]. Yet, during the course of many diseases the disturbance of this homeostasis occurs as part of the pathogenesis. To reestablish the PC homeostasis the bile acid phospholipid conjugate ursodeoxycholyl lysophosphatidylethanolamide (UDCA-LPE) was developed and synthesized for liver targeted supplementation of the PC precursor LPE [12]. UDCA-LPE exerted a greater anti-inflammatory and anti-apoptotic effect in TNF-α stressed hepatocytes exceeding the effect of the individual components [12, 13]. Furthermore, UDC-LPE demonstrated protective effects in endotoxin-mediated acute liver failure and NASH mouse models [13, 14].

Previous studies have demonstrated that PC inhibits LPS-mediated multi-organ failure [15] and directly exerts an anti-inflammatory effect on LPS-stimulated KC [16, 17]. Additionally, PC has been shown to reduce the activation and profibrogenic reaction of HSC [18]. The purpose of this study was to investigate if UDCA-LPE has a direct anti-inflammatory effect on LPS-stimulated KC and to test if the modulation of the KC driven inflammation also attenuates the profibrogenic activation of HSC.

## Results

### UDCA-LPE ameliorates LPS-induced inflammation

**Fig 1:**
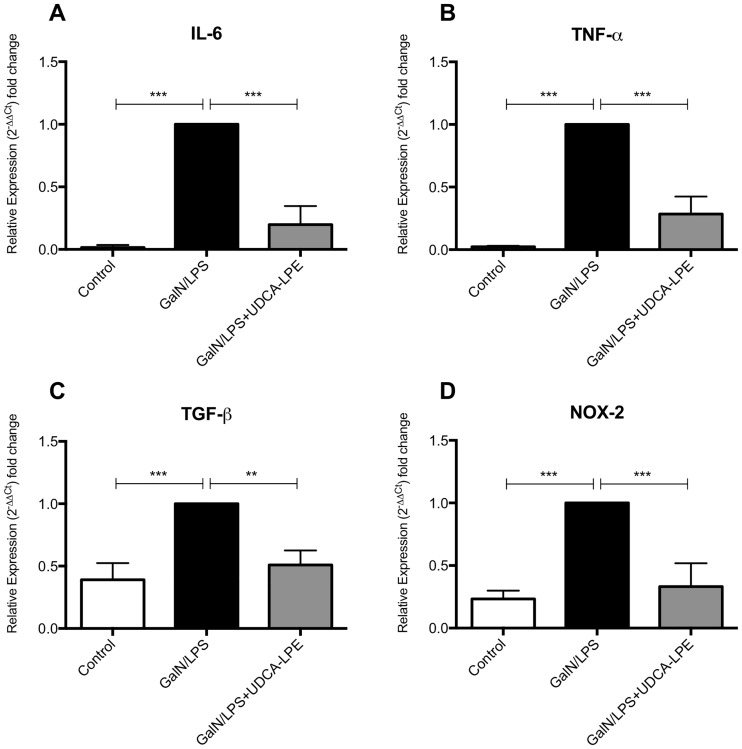
UDCA-LPE inhibits GalN/LPS-induced liver inflammation *in vivo*. C57BL/6 mice were injected i.p. with GalN at 700 mg/kg together with LPS at 10 ng/kg with or without 1 h pretreatment with UDCA-LPE at 30 mg/kg. mRNA Expression levels of IL-6 (A), TNF-α (B), TGF-β (C) und NOX-2 (D) in liver tissue of mice after 5 h of GalN/LPS exposure were quantified by qRT-PCR. Bars represent mean values ±SD. Each experiment was performed in triplicates. *** p < 0.001; ** p < 0.01.

First, the anti-inflammatory potential of UDCA-LPE was tested *in vivo* after i.p. injection of GalN ⁄LPS. Exposure towards the endotoxin resulted in a pronounced increase of IL-6, TNF-α, TGF-β and NOX-2 expression levels (Fig 1A–1D). Compared to untreated animals, the greatest upregulation was observed for the pro-inflammatory cytokines IL-6 (62.2x) and TNF-α (43.3x) followed by NOX-2 (4.2x) and TGF-β (2.6x). Treatment with UDCA-LPE greatly reduced expression levels by 80.2%, 71.5%, 66.8% and 49.0% for IL-6, TNF-α, NOX-2 and TGF-β respectively.

**Fig 2:**
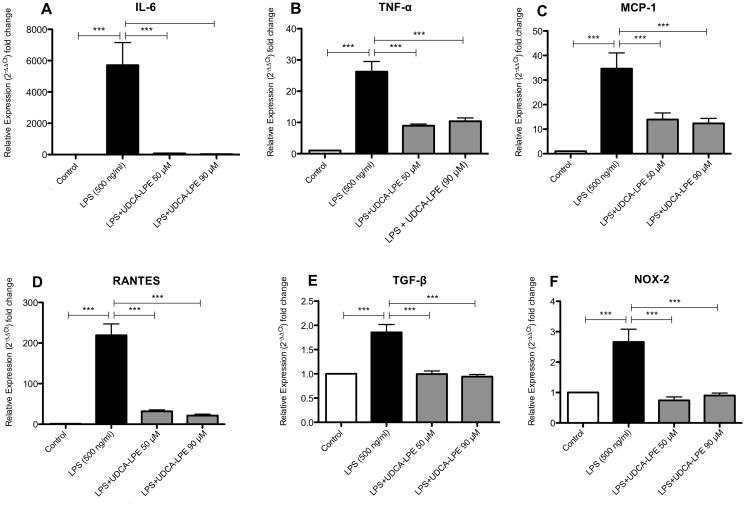
UDCA-LPE inhibits LPS-induced inflammation in RAW264.7 cells. RAW264.7 cells were treated with LPS (500 ng/ml) ± UDCA-LPE (50/90 μM) *in vitro*. mRNA expression levels of IL-6 (A), TNF-α (B), MCP-1 (C), RANTES, (D) TGF-β (E) und NOX-2 (F) were quantified by qRT-PCR. Bars represent mean values ±SD. Each experiment was performed in triplicates. P-values: *** p < 0.001.

LPS initiates an inflammatory response mainly by activating Kupffer cells [19, 20]. Thus, the direct inhibitory effect on RAW 264.7 cells as an *in vitro* surrogate model for Kupffer cells [17, 21] was further investigated. Treatment with 500 ng/ml LPS for 4 h markedly increased the expression of the pro-inflammatory cytokines IL-6 and TNF-α by up to 5712 and 26.3 times respectively (Fig 2A and 2B). Comparable observations were made for the chemokines MCP-1 and RANTES (Fig 2C and 2D), whereas the increase of TGF-β and NOX-2 was considerably lower (Fig 2E and 2F). Treatment with 50 and 90 μM UDCA-LPE greatly decreased the expression of the aforementioned cytokines, chemokines, and NOX-2 demonstrating the direct anti-inflammatory effect of UDCA-LPE on the macrophage cell line RAW 264.7 (Fig 2A–2F). Evaluation of the anti-inflammatory effect of UDCA-LPE in murine primary Kupffer cells showed a significant LPS-induced increase of IL-6, TNF-α and MCP-1 with a significant reduction of IL-6, TNF-α expression by UDCA-LPE (S1 Fig).

Analysis of IL-6 secretion into the supernatant of LPS-exposed RAW cell showed a 155 fold increase of IL-6 compared to control cells. In contrast, treatment with UDCA-LPE 50 μM and 90 μM decreased IL-6 secretion by 88.5% and 89.6% respectively (S2 Fig). Viability assessment of RAW264.7 cells after treatment with UDCA-LPE did not reveal direct cell toxicity as the cause for reduced IL-6 secretion (S3 Fig).

### UDCA-LPE downregulates the TLR4 pathway

**Fig 3:**
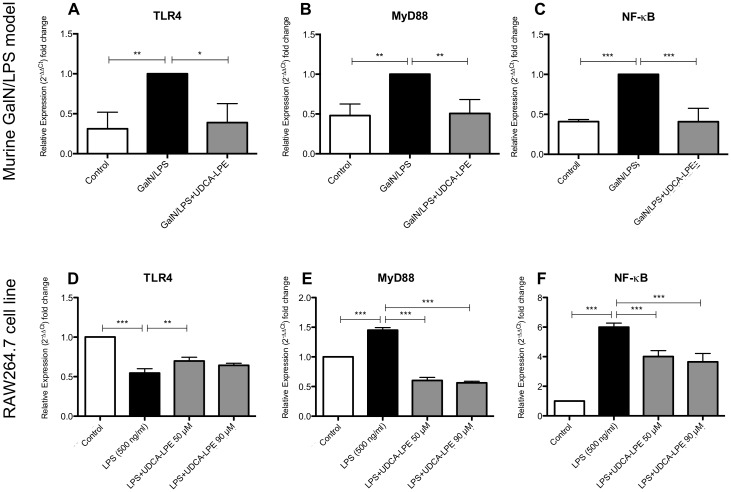
UDCA-LPE downregulates the TLR4-pathway *in vivo* and *in vitro*. mRNA expression levels of TLR4, MyD88 and NF-kB in the inflammatory acute liver failure GalN/LPS mouse model (A-C) and in LPS exposed (500 ng/ml; 4h) RAW264.7 cells (D-F) and when treated with UDCA-LPE are illustrated. Bars represent mean values ±SD. Each experiment was performed in triplicates. P-values: *** p < 0.001; ** p < 0.01; * p < 0.05.

In order to initiate a pro-inflammatory reaction LPS binds to TLR4 on the cell surface which leads to the activation of its downstream signaling cascade including the key modulators MyD88 and NF-kB. To further investigate the underlying mechanisms of UDCA-LPE acting as an anti-inflammatory compound *in vivo* and *in vitro*, we analyzed the expression profile of TLR4, MyD88 and NF-kB in the GalN/LPS model as well as in RAW264.7 cells. In GalN/LPS whole liver samples TLR4, MyD88, and NF-kB were greatly induced whereas treatment with UDCA-LPE was capable of completely abrogating the induction of these molecules (Fig 3A–3C). In comparison, LPS treatment of RAW264.7 cells led to a significant decrease of TLR4 while significantly inducing MyD88 and NF-kB. Interestingly, UDCA-LPE reversed TLR4 downregulation induced by LPS to a small extent and led to a significant decrease of elevated MyD88 and NF-kB expression levels (Fig 3D and 3E). In summary, UDCA-LPE mostly reversed the activation of the TLR4 pathway as part of its anti-inflammatory activity.

### UDCA-LPE modulates nitric oxide production

**Fig 4:**
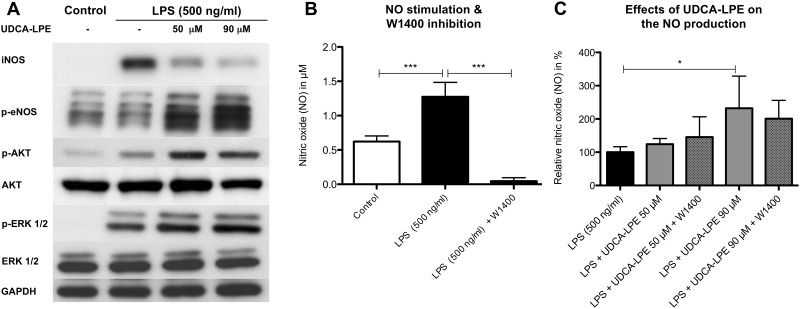
UDCA-LPE mediates iNOS-independent NO production in LPS stimulated RAW 264.7 macrophages. A) Western Blot analysis: iNOS (4h), phosphorylation of eNOS (Ser 1177) (2h) and AKT & ERK1/2 (30 min.) in RAW264.7 cells treated with LPS (500 ng/ml) ± UDCA-LPE (50/90 μM). B & C) Nitric oxide quantification after 6h via the Griess reaction in LPS (500 ng/ml) ± UDCA-LPE (50/90 μM) treated RAW 264.7 cells. iNOS inhibition was performed with μM 25 of W1400. All experiments have been performed in triplicates or quadruplets. Representative western blots are shown. Bars represent mean values ±SD. P-values: *** p < 0.001; * p < 0.05.

In Kupffer cells LPS also induces the inducible nitric oxide synthase (iNOS) resulting in nitric oxide (NO) production as part of the inflammatory reaction. Since the induction of iNOS is mediated via NF-kB which was lowered by UDCA-LPE, we analyzed iNOS protein content in RAW264.7 cells Exposure towards LPS for 4h pronouncedly increased iNOS levels, which could be reduced by 64% and 73% due to treatment with 50 μM and 90 μM UDCA-LPE respectively (Fig 4A). Notably, despite the decrease of iNOS, NO levels were not reduced by UDCA-LPE and even increased significantly to up to 200% when cells were treated with 90 μM UDCA-LPE compared to LPS treatment alone (Fig 4C). UDCA-LPE-induced NO increase was further confirmed to be iNOS-independent by blocking iNOS with the selective inhibitor W1400 (Fig 4B and 4C).

To further elucidate the source of NO after treatment with UDCA-LPE, phosphorylation status of the endothelial nitric oxide synthase (eNOS) was evaluated. The results showed a 2.6 and 3.8 time increase of phosphorylated eNOS due to 50 and 90 μM UDCA-LPE respectively. Western blot analysis revealed that, similar to UDCA, UDCA-LPE is capable of increasing cellular protein expression of eNOS (S4 Fig). Since both AKT and ERK1/2 are part of the TLR4 pathway and are able to activate eNOS, the phosphorylation status upon exposure towards LPS ± UDCA-LPE was analyzed (Fig 4A). The results showed that pAKT and pERK1/2 significantly increased in RAW 264.7 cells treated with UDCA-LPE compared to cells after LPS stimulation alone.

### UDCA-LPE modulates hepatic stellate cell activation in a pro-inflammatory environment

**Fig 5:**
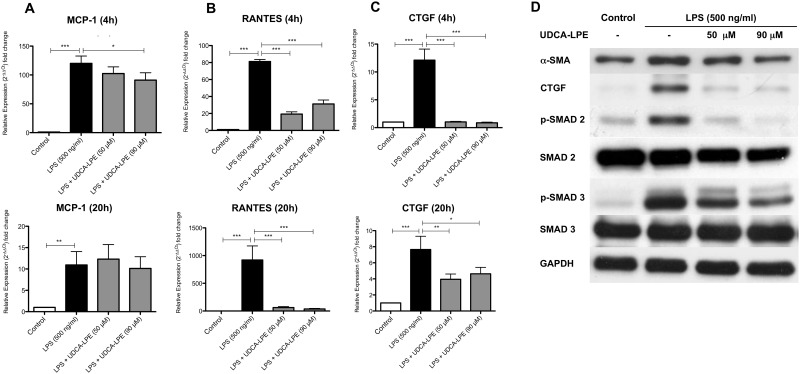
UDCA-LPE limits the pro-fibrogenic and pro-inflammatory activation of HSC. Primary hepatic stellate cells were incubated with conditioned supernatant from LPS (500 ng/ml) ± UDCA-LPE (50/90 μM) treated RAW264.7 macrophages for 20h. A-C) mRNA expression levels of MCP-1, RANTES and CTGF after 4 & 20 hours were quantified by qRT-PCR. D) Representative western blot expression profiles of α-SMA (20h), CTGF (4h), (p-)SMAD 2 (Ser 465/467) (1h) and (p-)SMAD 3 (Ser 423/425) (4h). All experiments have been performed in triplicates or quadruplets. Bars represent mean values ±SD. P-values: *** p < 0.001; ** p < 0.01; * p < 0.05.

By liberating a variety of pro-inflammatory mediators after LPS stimulation, KC activate quiescent HSC which in return contributes to the inflammatory cascade by secreting e.g. chemokines to recruit further immune cells and turn into a profibrogenic subtype [22, 23]. Thus, we firstly analyzed UDCA-LPE for its potential to limit chemokine secretion in HSC. Conditioned medium from LPS-stimulated RAW264.7 cells led to a distinct increase of MCP1 and RANTES expression in HSC. In contrast, treatment with UDCA-LPE resulted in a considerable inhibition of RANTES mRNA levels at both tested time points, whereas reduction of MCP-1 was less pronounced but significant after 4h for 90 μM UDCA-LPE (Fig 5A and 5B).

Subsequently we investigated whether UDCA-LPE treatment of LPS stimulated RAW264.7 cells would also have an impact on the activation status of HSC. α-smooth muscle actin (α-SMA), a marker for activated HSC, increased when cells were treated with conditioned medium from LPS-stimulated RAW264.7 cells. Treatment with UDCA-LPE led to a significant and concentration-dependent decrease of α-SMA protein levels by 18.2% and 42.2% respectively (Fig 5D). Similarly, CTGF, an important pro-fibrogenic modulator of liver fibrosis, increased after 4h and 20h in HSC cultured in an inflammatory environment whereas treatment with conditioned medium from LPS + UDCA-LPE treated RAW264.7 cells resulted in very limited and significantly lower CTGF expression and protein levels (Fig 5C and 5D). One of the central regulators of CTGF expression is TGFβ via the SMAD2/3 pathway. Comparable to CTGF expression levels phosphorylated SMAD2 and SMAD3 increased in LPS-stimulated HSC while it significantly decreased in HSC treated with conditioned medium from LPS + UDCA-LPE cultured RAW264 cells (Fig 5D). Additionally, treatment with 90 μM UDCA-LPE also decreased the protein expression of unphosphorylated SMAD2 by up to 36.7%. In summary, the inhibitory influence of UDC-LPE on the pro-inflammatory response of RAW264.7 cells towards LPS resulted in limited HSC activation and presumably limited chemotaxis of immune cells by lowering chemokine expression.

### UDCA-LPE impairs IL-6-mediated CTGF induction

**Fig 6:**
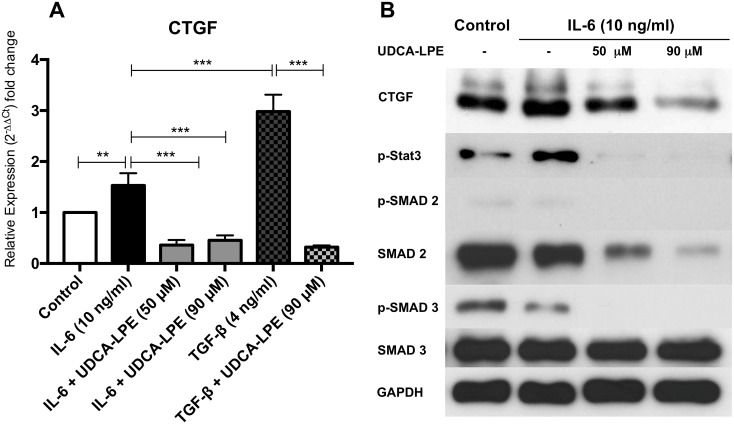
UDCA-LPE inhibits IL-6 mediated CTGF induction in HSC. A) CTGF mRNA expression of HSC treated with IL-6 (10 ng/ml), TGF-β (4 ng/ml) ± UDCA-LPE (50/90 μM) were quantified by qRT-PCR. B) Western blot analysis of HSC treated with IL-6 (10 ng/ml) ± UDCA-LPE (50/90 μM): CTGF (20h), Stat-3 (1h), (p-)SMAD 2 (Ser 465/467) & (p-)SMAD 3 (Ser 423/425) (1h). All experiments have been performed in triplicates or quadruplets. Representative Western Blots are shown. Bars represent mean values ±SD. p-values: *** p < 0.001; ** p < 0.01.

The CTGF promoter region also contains a domain for phosphorylated Stat3 [24] suggesting that IL-6 may also modulate CTGF expression. Since both IL-6 and CTGF were markedly reduced due to UDCA-LPE, we investigated the influence of IL-6 on CTGF induction and the ability of UDCA-LPE to modify this signaling pathway. Notably, our results showed induction of CTGF by IL-6, albeit less powerful than TGF-β, whereas treatment with UDCA-LPE significantly reduced CTGF expression even below the level of untreated cells in all tested settings (Fig 6A). Exposure towards IL-6 resulted in phosphorylation of Stat3, which was markedly suppressed by UDCA-LPE even below baseline levels. Downregulation of CTGF was further accompanied by lower levels of pSMAD2 and pSMAD3 in HSC exposed to the supernatant of UDCA-LPE-treated cells (Fig 6B).

## Discussion

Many acute and chronic liver diseases of various etiologies are accompanied or even mainly driven by an inflammatory reaction initiated by KC via the activation of the TLR4 pathway leading to parenchymal damage, liver fibrosis, and eventually to liver failure [3, 4, 8, 19, 20, 25]. Thus, the pivotal relevance of KC driven inflammation during the course of many disease makes them an appealing target for therapy.

Previous investigations on the synthetic bile acid-phospholipid conjugate UDCA-LPE as a therapeutic agent revealed considerable hepatoprotective and anti-inflammatory effects in experimental models of acute liver injury, non-alcoholic fatty liver diseases as well as hepatic ischemia and reperfusion injury [1, 2, 13, 14, 26]. In this study, we further characterized the anti-inflammatory potential of UDCA-LPE *in vivo* and *in vitro* and investigated how the inhibition of inflammation impacts the pro-fibrogenic and pro-inflammatory response of hepatic stellate cells. As illustrated above, UDCA-LPE demonstrated potent anti-inflammatory properties *in vivo* in a mouse model for acute liver failure as well as in the macrophage cell line RAW264.7 by directly downregulating the TLR4 pathway. Moreover, modulation of the inflammatory environment of LPS-stimulated RAW264.7 cells by UDCA-LPE resulted in a lower pro-fibrogenic activation status and a impaired pro-inflammatory response of HSC.

It is well recognized that TNF-α is one of the key mediators in endotoxemic acute liver failure [27, 28]. Besides a direct pro-apoptotic effect on hepatocytes, it aggravates the inflammatory reaction by additionally stimulating the production of a myriad of mediators leading to tissue hypoxia, reactive oxygen production and to the amplification of chemokine-dependent immune cell recruitment [20, 29, 30]. Additionally, beyond the liver, TNF-α spill-over induces a systemic inflammatory response resulting in a multi-organ failure worsening prognosis [20, 27]. Specific inhibition of TNF-α [28], as well as other cytokines and chemokines [31, 32] in the setting of acute liver failure have shown to ameliorate inflammation and liver damage. Thus, downregulation of these mediators may contribute to the hepatoprotective effects of UDCA-LPE. On the other hand, IL-6, TNF-α and TGF-β are also known to contribute to liver regeneration [33, 34]. However, UDCA-LPE activates hepatocyte proliferation directly by activation of the PI3K/AKT signaling pathway so that a lower level of cytokine-induced proliferation may be compensated directly by UDCA-LPE-mediated regenerative effects in the setting of experimental acute liver failure [12].

Similar to acute liver failure, inflammatory mediators such as IL-6, TNF-α, TGF-β [35], MCP-1 and RANTES [23, 36] are crucially involved in the pathogenesis of NASH including insulin resistance, steatosis and apoptosis of hepatocytes, immune cell infiltration, and liver fibrosis all of which could be greatly ameliorated by UDCA-LPE accompanied by a downregulation of inflammatory mediators including MCP-1 and TNF-α [14]. Since KC are mainly responsible for TNF-α and MCP-1 secretion in the liver [37] and as UDCA-LPE had an anti-inflammatory effect on LPS-stimulated RAW264 cells, it may be assumed that UDCA-LPE directly exerts its anti-inflammatory potential on KC in acute and chronic liver diseases.

Anti-inflammatory properties of UDCA-LPE may reflect the ability of the conjugate to directly downregulate crucial TLR4 pathway molecules including MyD88 and NF-kB as this has been demonstrated to be an effective way to limit KC-mediated inflammation [4]. Interestingly, TLR4 was already downregulated in RAW 264.7 cells by LPS treatment alone which is assumed to be part of the physiological countermeasure to limit inflammatory response in KC [38]. Notably, despite the inhibition of crucial TLR4 pathway components, other associated signaling molecules such as AKT and ERK1/2 were even activated by UDCA-LPE. Thus, further studies on the influence of UDCA-LPE on TLR4 pathway activity and its interaction with other signaling pathways is needed to understand the anti-inflammatory profile of UDCA-LPE.

UDCA-LPE greatly reduced LPS-mediated iNOS expression in RAW264.7 cells. Against expectations, UDCA-LPE even increased NO production in an iNOS-independent manner, which was accompanied by activated AKT and ERK1/2 in RAW264.7 cells. AKT and ERK1/2 have been shown to induce eNOS leading to an increase of NO production [12, 39]. In the setting of endotoxemia, NO has the potential to inhibit hepatocellular apoptosis and inflammation [40]. However, in the presence of reactive oxygen species (ROS) mainly produced by NOX-2 in KC, NO reacts with ROS to cytotoxic peroxynitrites counteracting the protective effects of NO. Since NOX-2 expression was decreased in UDCA-LPE-treated RAW264.7 cells and the NO production is increased, the overall ratio of NO to peroxynitrite favors NO which is believed to be hepatoprotective [40, 41].

Activation of HSC is a central step in the pathophysiology of hepatic fibrogenesis. KC thereby play a crucial role by secreting several activating mediators including TNF-α, TGF-β, MCP-1 and RANTES [42]. Conditioned medium from LPS-stimulated RAW 264.7 cells led to an increased expression of the HSC activation marker α-SMA, which could be prevented by concomitant treatment with UDCA-LPE. Further investigation revealed that phosphorylation of SMAD 2 and 3, known to be crucially involved in the TGF-β pathway was reduced in the UDCA-LPE treatment group [43]. Previous studies on TGF-β had already shown a direct inhibitory effect of UDCA-LPE on the TGF-β/SMAD2/3 pathway [13]. It is therefore likely that not only the reduced expression of inflammatory mediators but also remaining UDCA-LPE in the supernatant may be responsible for reduced of HSC activation. Interestingly, the spontaneous activation of HSC, which usually occurs when cultured on uncoated plastic could not be reversed by UDCA-LPE similar to the suppression of the SMAD2/3 molecules [43].

CTGF stimulation of HSC in the pro-inflammatory environment of conditioned medium from LPS stimulated macrophages was markedly higher compared to treatment with TGF-β alone implying that other factors may be involved in CTGF induction. Previous studies in cardiomyocytes demonstrated the potential of IL-6 induced CTGF expression [24] whereas the knockout of Stat3 in HSC has been reported to be associated with reduced CTGF expression [44]. As expected, IL-6 treatment alone induced CTGF expression in HSC via phosphorylation of the downstream mediator Stat-3 which is known to have a binding site at the promoter region of CTGF [24]. In summary, UDCA-LPE can limit CTGF-expression in HSC by reducing the expression of TGF-β and IL-6 as well as by inhibition of their intracellular pathways leading to CTGF induction.

Although expected, an increase in collagen expression by HSC was not observed in the inflammatory setting used despite the activated SMAD 2/3 pathway. It may be assumed that the used model rather mimics acute than chronic inflammation and that the myriad of mediators counteracts collagen expression as it has been reported e.g. for TNF-α [45]. Nonetheless, in light of the fact that knockdown of CTGF significantly reduced experimental liver fibrosis and that UDCA-LPE reduced CTGF expression even below the level of untreated control cells may partly explain the overall anti-fibrotic effects of UDCA-LPE [2, 14, 44, 46].

When exposed to an inflammatory environment HSC are stimulated by pro-inflammatory cytokines such as e.g. IL-6 and TNF-α to secrete the chemokines MCP-1 and RANTES similar to KC in order to participate in the recruitment of further immune and hepatic stellate cells to the site of inflammation [22, 23]. Although downregulation of RANTES was more pronounced compared to MCP-1, an overall reduction of chemokine expression in RAW 264.7 cells and HSC was observed and may have similar limiting effects on HSC recruitment, proliferation and collagen deposition as direct blocking of RANTES and MCP-1 [22, 23, 36, 47].

In conclusion, the bile acid-phospholipid conjugate UDCA-LPE exerts direct anti-inflammatory effects on the KC surrogate cell line RAW264.7, which resulted in an impaired activation of HSC. Along with the previously reported direct hepatoprotective and anti-fibrotic effects of UDCA-LPE, the findings imply a promising potential for the drug candidate as an experimental approach for the treatment of acute and chronic liver diseases.

## Supporting information

S1 Fig UDCA-LPE inhibits LPS induced inflammation in primary Kupffer cells. (TIFF)

S2 Fig UDCA-LPE greatly decreases the LPS induced IL-6 secretion. (TIFF)

S3 Fig UDCA-LPE has no negative impact on the viability of RAW264.7. (TIFF)

S4 Fig UDCA-LPE induces eNOS expression in RAW264.7 cells. (TIFF)

S1 Table List of used primary western blot antibodies. (DOCX)

S2 Table List of used secondary western blot antibodies. (DOCX)

S3 Table List of measured genes Assay ID. (DOCX)
