# Supplementary figures and images for: Anti-inflammatory properties of ursodeoxycholyl lysophosphatidylethanolamide in endotoxin-mediated inflammatory liver injury
Source: PLoS One. 2018 May 24;13(5):e0197836. doi: 10.1371/journal.pone.0197836 (PMC5967712; doi:10.1371/journal.pone.0197836)

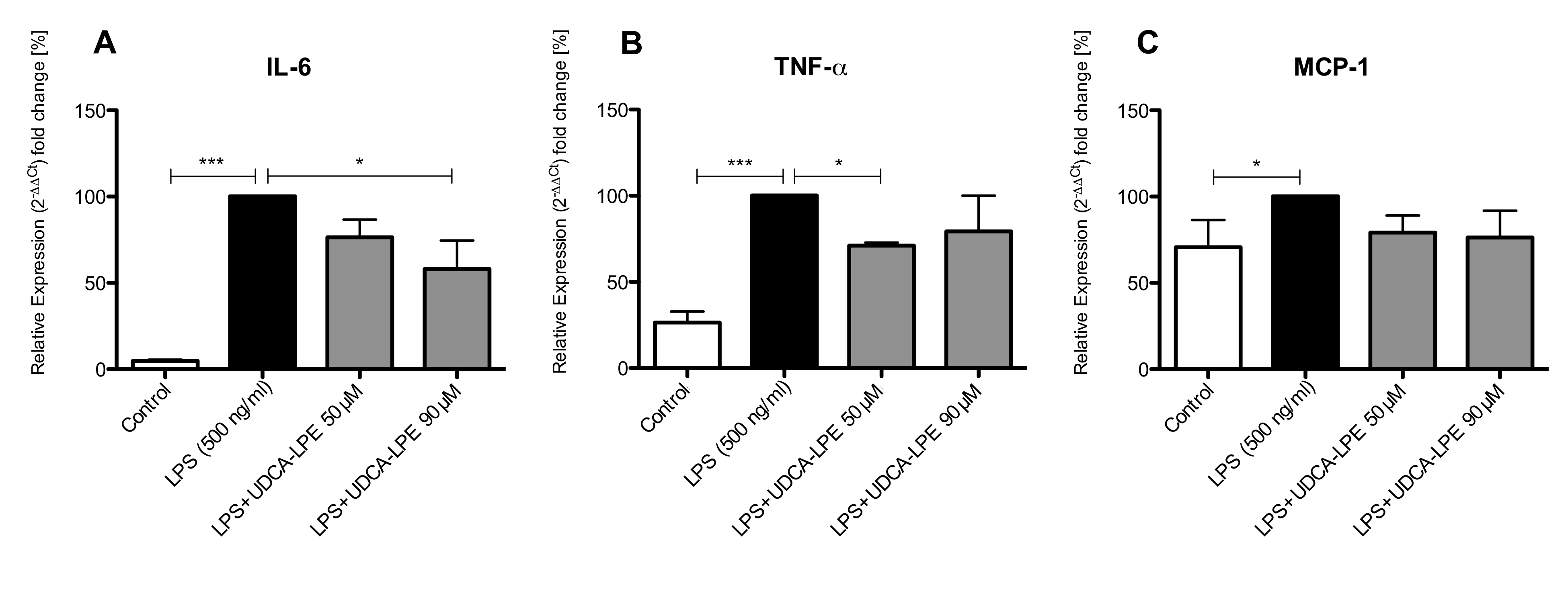

Supplement: S1 Fig — (TIFF) [file pone.0197836.s001.tiff]

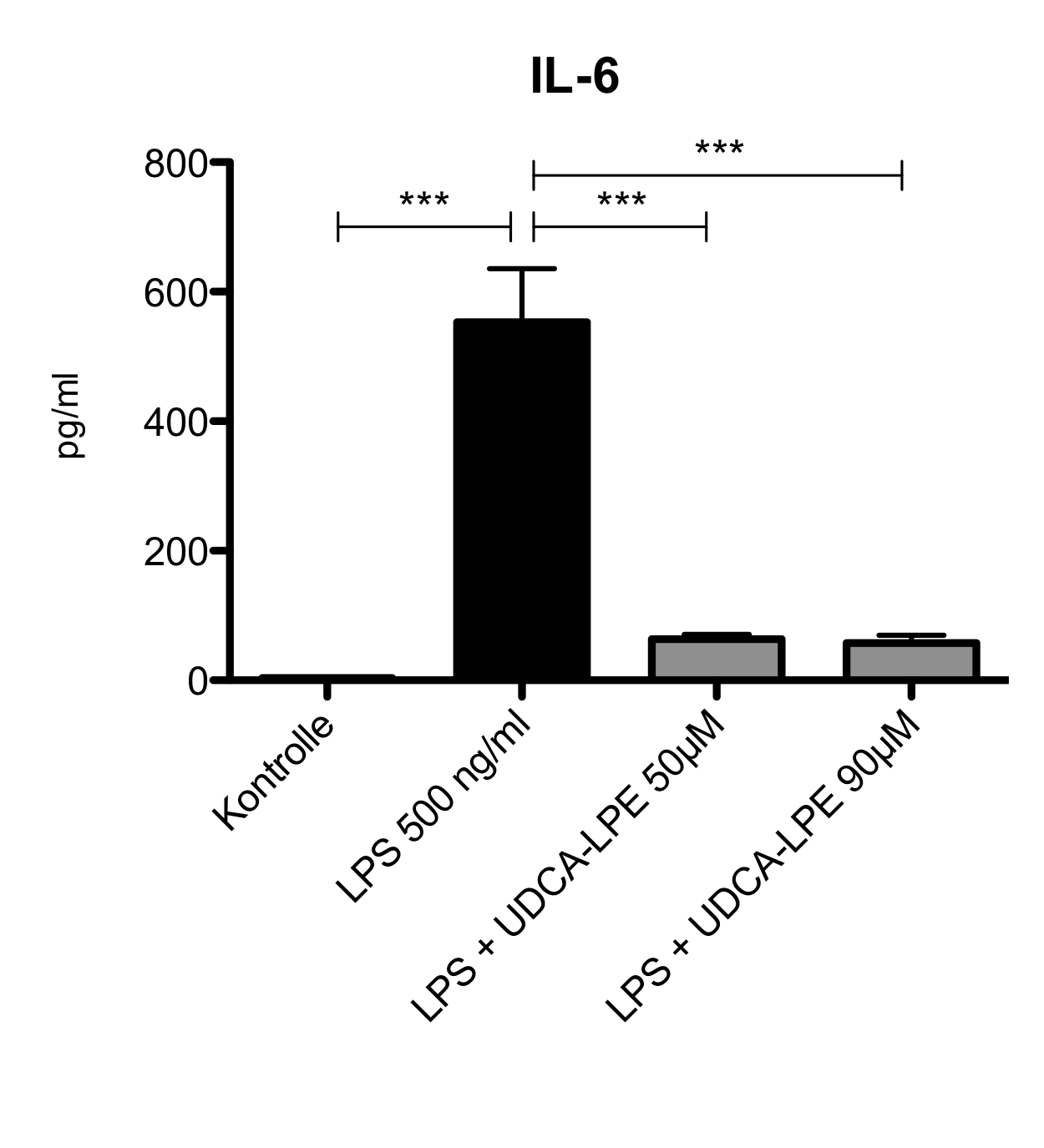

Supplement: S2 Fig — (TIFF) [file pone.0197836.s002.tiff]

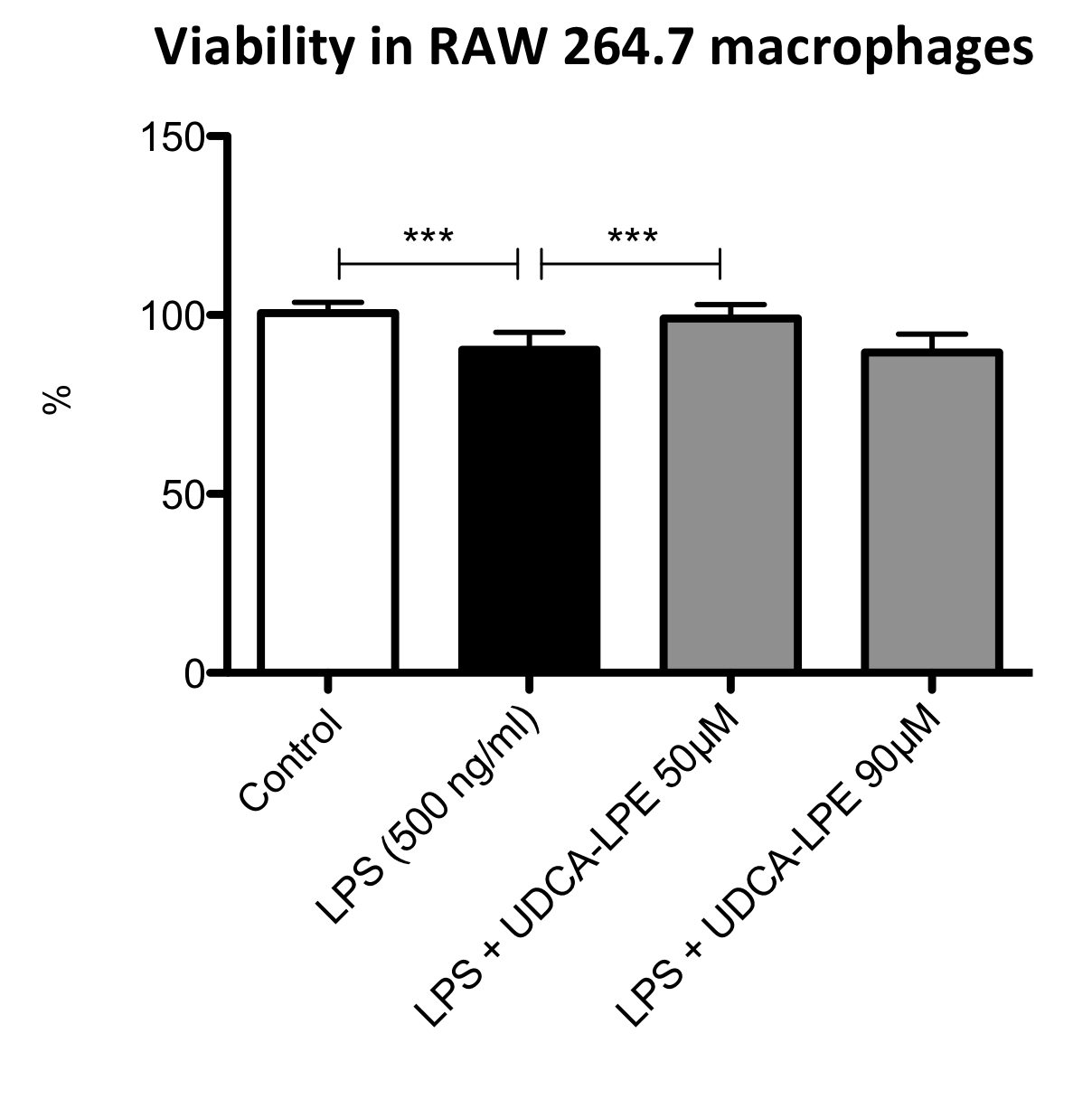

Supplement: S3 Fig — (TIFF) [file pone.0197836.s003.tiff]

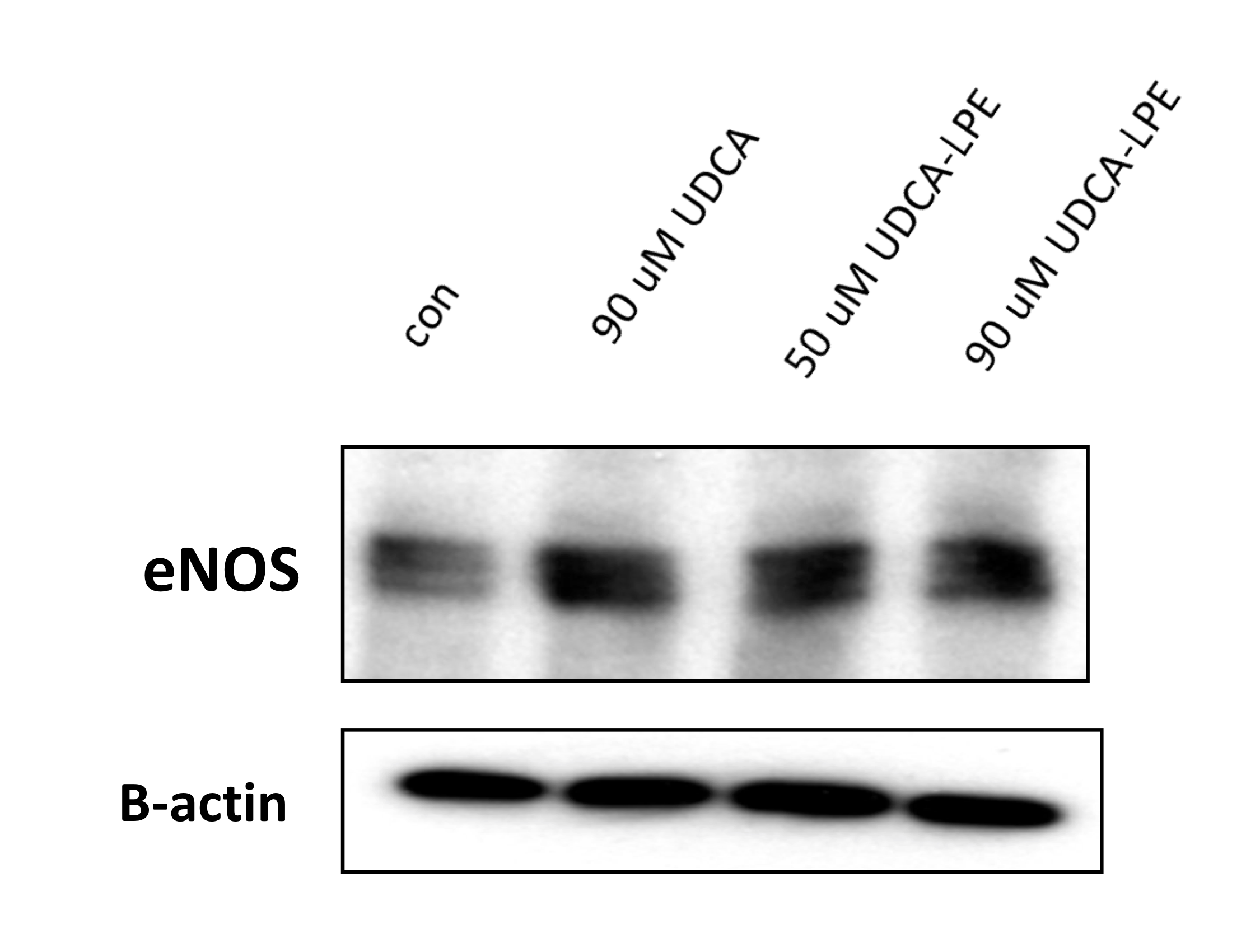

Supplement: S4 Fig — (TIFF) [file pone.0197836.s004.tiff]
