# Supplementary material for: Anti-inflammatory properties of ursodeoxycholyl lysophosphatidylethanolamide in endotoxin-mediated inflammatory liver injury
Source: PLoS One. 2018 May 24;13(5):e0197836. doi: 10.1371/journal.pone.0197836 (PMC5967712; doi:10.1371/journal.pone.0197836)
Supplement: S1 Table — (DOCX) [file pone.0197836.s005.docx]

**S1 Table: List of used primary western blot antibodies**

| **Antigen** | **Antibody-dilution** | **Species** | **Company** |
| --- | --- | --- | --- |
| AKT | 1:2000 | Mouse | BioSource Int. Inc., USA |
| p-AKT (Ser 473) | 1:2000 | Mouse | Cell Signaling^®^, UK |
| α-SMA (Smooth Muscle) | 1:50.000 | Rabbit | Epitomics^®^, USA |
| β-Tubulin | 1:20.000-1:100.000 | Rabbit | Epitomics^®^, USA |
| CTGF | 1:1000 | Goat | Santa Cruz Biotechnology Inc., USA |
| eNOS | 1:2000 | Rabbit | Cell Signaling^®^, UK |
| p-eNOS (Ser1177) | 1:3000 | Rabbit | Cell Signaling^®^, UK |
| GAPDH | 1:50.000 | Rabbit | Cell Signaling^®^, UK |
| p44/42 MAPK (Erk1/2) | 1:10.000 | Rabbit | Cell Signaling^®^, UK |
| Phospho-p44/42 MAPK (Erk1/2) (Thr202/Tyr204) | 1:1000 | Mouse | Cell Signaling^®^, UK |
| iNOS | 1:3000 | Mouse | BD Biosciences, USA |
| NF-κB p65 | 1:5000 | Rabbit | Epitomics^®^, USA |
| SMAD 2 | 1:2000 | Rabbit | Epitomics^®^, USA |
| p-SMAD 2 (Ser465/467) | 1:1000-1:10.000 | Rabbit | Cell Signaling^®^, UK |
| SMAD 3 | 1:1000 | Rabbit | Epitomics^®^, USA |
| p-SMAD 3 (Ser42325) | 1:1000-1:10.000 | Rabbit | Epitomics^®^, USA |
| p-Stat3 (Tyr705) | 1:4000 | mouse | Cell Signaling^®^, UK |
