# Supplementary material for: Anti-inflammatory properties of ursodeoxycholyl lysophosphatidylethanolamide in endotoxin-mediated inflammatory liver injury
Source: PLoS One. 2018 May 24;13(5):e0197836. doi: 10.1371/journal.pone.0197836 (PMC5967712; doi:10.1371/journal.pone.0197836)
Supplement: S2 Table — (DOCX) [file pone.0197836.s006.docx]

**S2 Table: List of used secondary western blot antibodies**

| **Antigen** | **Antibody-dilution** | **Species** | **Company** |
| --- | --- | --- | --- |
| Anti-mouse IgG | 1:10.000-1:20.000 | Goat | Santa Cruz Biotechnology Inc., USA |
| Anti-rabbit IgG | 1:5.000-1:50.000 | Goat | Epitomics^®^, USA |
| Anti-goat IgG | 1:10.000 | Donkey | Santa Cruz Biotechnology Inc., USA |
