# Supplementary material for: Anti-inflammatory properties of ursodeoxycholyl lysophosphatidylethanolamide in endotoxin-mediated inflammatory liver injury
Source: PLoS One. 2018 May 24;13(5):e0197836. doi: 10.1371/journal.pone.0197836 (PMC5967712; doi:10.1371/journal.pone.0197836)
Supplement: S3 Table — (DOCX) [file pone.0197836.s007.docx]

**S3 Table:** List of measured genes Assay ID

| **Species** | **Gene symbol** | **Assay ID** | **Gene name** |
| --- | --- | --- | --- |
| ***Homo Sapiens***  ***Mus Musculus*** | CCL2 | Hs00234140_m1 | Monocyte Chemotactic Protein-1 (MCP-1) |
|  | CCL5 | Hs00982282_m1 | Regulated on Activation, Normal T cell Expressed and Secreted (RANTES) |
|  | CTGF | Hs00170014_m1 | Connective Tissue Growth Factor (CTGF) |
|  | GAPDH | Hs02758991_g1 | Glyceraldehyde-3- phosphate dehydrogenase (GAPDH) |
|  |  |  |  |
|  | CCL2 | Mm00441242_m1 | Monocyte Chemotactic Protein-1 (MCP-1) |
|  | CCl5 | Mm01302428_m1 | Regulated on Activation, Normal T cell Expressed and Secreted (RANTES) |
|  | GAPDH | Mm99999915_g1 | Glyceraldehyde-3- phosphate dehydrogenase (GAPDH) |
|  | IL6 | Mm012010733_m1 | Interleukin-6 (IL-6) |
|  | MYd88 | Mm00440338_m1 | Myeloid differentiation primary response gene (88) (MYD88) |
|  | NF-κB | Mm00476379_m1 | Nuclear factor kappa-light-chain-enhancer of activated B cells (NF-κB) |
|  | Cybb | Mm01287743_m1 | Nicotinamide adenine dinucleotide phosphate-oxidase 2 (NOX-2) |
|  | Tlr4 | Mm00445273_m1 | Toll-like Receptor 4 (TLR4) |
|  | tnf | Mm00443256_m1 | Tumor Necrosis Factor-α (TNF-α) |
|  | Tgfb1 | Mm01178820_m1 | Transforming Growth Factor β 1 (TGF-β) |
